# Analytical and computational modelling for wave energy systems: the example of oscillating wave surge converters

**DOI:** 10.1007/s10409-017-0683-6

**Published:** 2017-06-07

**Authors:** Frédéric Dias, Emiliano Renzi, Sarah Gallagher, Dripta Sarkar, Yanji Wei, Thomas Abadie, Cathal Cummins, Ashkan Rafiee

**Affiliations:** 1 School of Mathematics and Statistics, University College Dublin, MaREI Centre, Belfield, Dublin 4, Ireland; 2 Department of Mathematical Sciences, Loughborough University, Leicestershire, LE11 3TU UK; 3 Research, Environment and Applications Division, Met Éireann, Glasnevin, Dublin 9, Ireland; 4 Department of Engineering Sciences, University of Oxford, Oxford, UK; 5 Advanced Production Engineering, University of Groningen, Groningen, The Netherlands; 6 Dublin City University, Glasnevin, Dublin 9 Ireland; 7 School of Engineering, University of Edinburgh, Edinburgh, UK; 8 Carnegie Clean Energy Limited, Northam, Australia

**Keywords:** Wave energy, Wave energy converter, Slamming, Wave resource

## Abstract

The development of new wave energy converters has shed light on a number of unanswered questions in fluid mechanics, but has also identified a number of new issues of importance for their future deployment. The main concerns relevant to the practical use of wave energy converters are sustainability, survivability, and maintainability. Of course, it is also necessary to maximize the capture per unit area of the structure as well as to minimize the cost. In this review, we consider some of the questions related to the topics of sustainability, survivability, and maintenance access, with respect to sea conditions, for generic wave energy converters with an emphasis on the oscillating wave surge converter. New analytical models that have been developed are a topic of particular discussion. It is also shown how existing numerical models have been pushed to their limits to provide answers to open questions relating to the operation and characteristics of wave energy converters.

## Introduction

Great prospects are offered by wave power devices for the marine renewable energy sector. However, no well-established wave energy industry is built anywhere in the world at present. Ireland has the potential to become a world-leading developer and manufacturer of the technologies that will enable the harnessing of ocean energy resources. Since 2013, Science Foundation Ireland (SFI) has funded the MaREI Centre, which is a cluster of university and industrial partners dedicated to solving scientific, technical, and socio-economic challenges across the marine and renewable energy sectors. Earlier, from 2011 to 2016, SFI supported a research project led by University College Dublin, which focused on sustainability, survivability, and maintainability for generic wave energy converters (WECs) with an emphasis on the oscillating wave surge converter (OWSC). The project was undertaken in partnership with Aquamarine Power Ltd. (APL), the company that developed the Oyster device. Unfortunately APL ceased to trade on 20 November 2015. However, the project shed light on a number of unanswered questions in fluid mechanics.

**Fig. 1:**
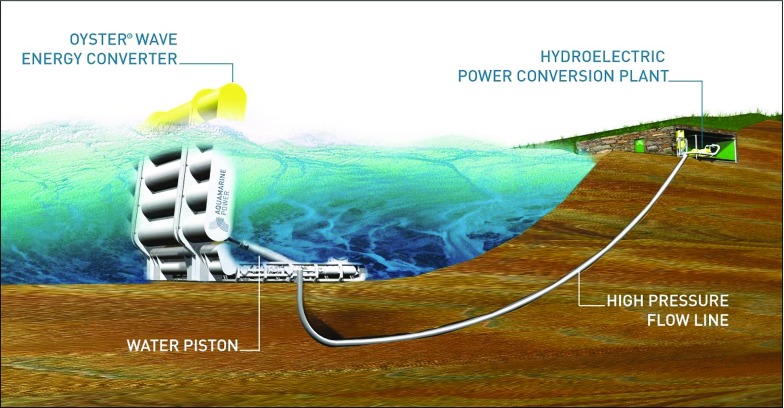
Artist’s sketch of the Oyster WEC concept

The aim of this review is to go through some of these unanswered questions and the solutions that our group based in University College Dublin provided over the period 2012–2016. These questions fall into three major themes, which range from local considerations for a single WEC, through considerations on an array of WECs to finally considerations on the global wave climate.

Issues addressed within these themes include wave impact and pressure loads on a single WEC, interaction between waves and a single WEC, viscous and nonlinear effects, slamming, device interactions for an array of WECs, optimal device spacing, wave climate prediction with improved coupling between wind modelling and wave modelling, and preferred geographical locations for nearshore WEC sites.

The main sections of the paper are devoted to the three major themes (hydrodynamics and loading of one WEC; arrays of WECs; wave climate). In Sect. 2, we address the survivability of ocean wave energy devices that are required to operate in a harsh and violent environment, such as the west coast of Ireland. They must be engineered in terms of their stability and structural strength to capture energy while operating under these extreme weather conditions. In Sect. 3, we address the efficiency of arrays of ocean wave energy devices. For wave energy to become commercially viable it is clear that WECs will have to be deployed in arrays. We developed novel tools to provide a better understanding of the behaviour of arrays of WECs. These tools allow an analysis of device interactions and of optimal device spacing for power production. In Sect. 4, we investigate the topic of wave climate from the perspective of wave energy, vital to address issues related to site selection, device and control specification, and access to maintenance of WECs. A detailed knowledge of the wave climate at the proposed deployment sites is necessary not only for the capture of energy but also for the maintenance of devices. Wave climate estimates rely largely on computer hindcast wind-wave models. We have used improved models to obtain accurate annual wave climate predictions in terms of significant wave height, direction and mean wave period, with a focus on the nearshore wave energy resource.

Our group has collaborated closely with APL on the development of the Oyster WEC. The Oyster WEC comprises a buoyant flap, hinged at the sea bed, whose pitching oscillations activate a set of double-acting hydraulic rams located on the seabed that pump high pressure fluid ashore via a sub-sea pipeline, as shown in Fig. 1. The fluid flow is converted into electric energy using a Pelton turbine. These bottom-hinged devices are intended for deployment in the nearshore environment, in relatively shallow water (ranging from 10 to 15 m). The Oyster WEC has a surface piercing flap that spans the entire water depth.

**Fig. 2:**
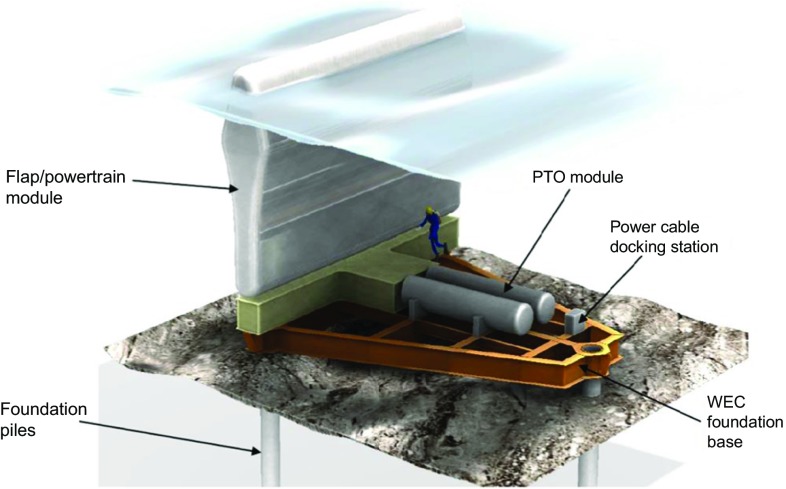
Schematic drawing of the WaveRoller WEC concept

Surface-piercing flap-type devices are designed to harvest wave energy in the nearshore environment. Established mathematical theories of wave energy conversion, such as 3-D point-absorber and 2-D terminator theories, have proved inadequate to accurately describe the behaviour of devices like Oyster, leading to distorted conclusions regarding the potential of such a concept to harness the power of ocean waves [1]. Accurate reproduction of the dynamics of Oyster required the introduction of a new reference mathematical model, the “flap-type absorber”. A flap-type absorber is a large thin device that extracts energy by pitching about a horizontal axis parallel to the ocean bottom. It is now accepted that the wave capture rate is best for a wide flap-type absorber [2] and the size of the flap drives, among other factors, the capital expenditure (CAPEX): more material increases the CAPEX. One of the difficulties that led to the failure of the Oyster WEC was its power take-off (PTO) system. The Finnish company AW-Energy is now working on the WaveRoller WEC [3], with a supposedly better PTO than that of the Oyster WEC (see Fig. 2). Interestingly, the first versions of the WaveRoller WEC were completely submerged, which was not optimal from the power capture perspective.

The governing equations for the hydrodynamics of WECs are the continuity and Navier–Stokes momentum equations with a free-surface. Typically, however, simplifications must be made to make the problem tractable. Such simplifications may be to consider only small perturbations to the free surface (linear waves), or to neglect the effects of viscosity by treating the fluid as an inviscid fluid. For the study of wave impact on structures, one also needs to incorporate equations for fluid-solid interactions. One of the challenges, then, is to know which effects are important for the physical phenomenon one wishes to describe. Standard computational fluid dynamics (CFD) tools are not suitable for farms of WECs, since typical CFD computations require several hours of CPU time for a single wave period. At present, they can only be useful at the local level, for example, to understand the loads and the viscous effects on a single WEC.

In the concluding Section, we will learn from the lessons of the past and give suggestions for the way forward.

## Survivability of wave energy systems

Early research and development studies of WECs focused mainly on floating devices like point- and line-absorbers [4]. Point absorbers are devices with dimensions that are much smaller than the incident wavelength (e.g., a heaving buoy), while line absorbers have one dominant horizontal dimension, with an order of magnitude that is at least one wavelength (e.g., an articulated raft) [5]. Line absorbers can work either as terminators or attenuators, depending on their alignment being, respectively, orthogonal or parallel to the direction of propagation of the incident waves. However, driven by the need for more powerful WECs to decrease energy production costs, the wave energy sector has evolved towards the design of new large-scale WECs, which do not belong to the point- and line-absorber categories, namely Oscillating Wave Surge Converters such as the Oyster or the WaveRoller devices. A key driver of the structural design of such devices is the pressure distribution and loading induced on the flap by the incident waves [1]. The pitch of an OWSC is driven by the strong exciting torque resulting from the pressure difference between its sides. Such a pressure difference originates because of the OWSC’s ability to favorably reflect, bend and shade waves in different areas of the surrounding sea. This diffractive dynamics is much stronger than that for point absorbers, and, therefore, requires a non-point-absorber explanation.

**Fig. 3:**
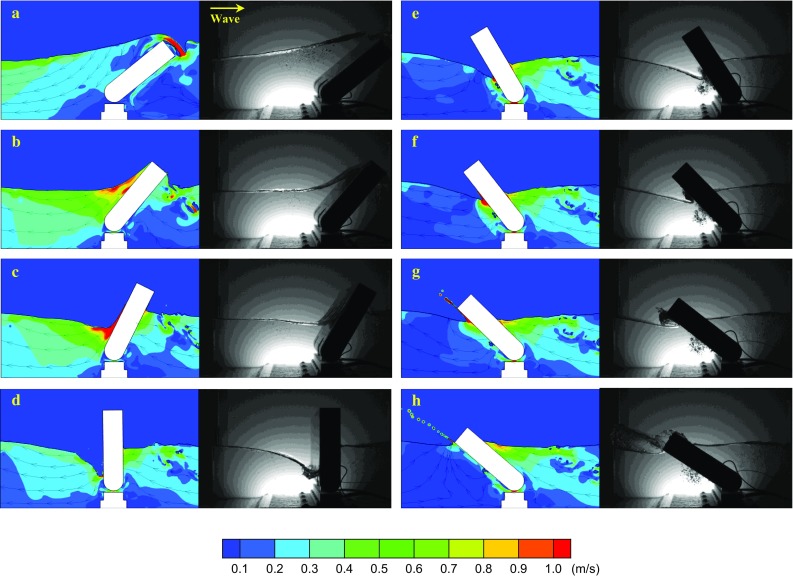
Typical slamming event of an OWSC observed both in numerical simulations (*left column*) and in laboratory experiments (*right column*). The wave propagates from left to right. The numerical results are colored by the velocity magnitude. The time difference between the first frame **a** and the last one **h** is 0.72 s. From Ref. [12]

Localised pressure points could compromise the structural integrity of the device if not identified and factored into the design. Details of pressure loads are, therefore, of great interest to the designer for both everyday wave climate conditions and in extreme/storm wave conditions. The latter has a much more significant effect on device failure [6]. As stated in Ref. [7], numerous coastal and marine structures are damaged by wave action each year. The damage is often caused by the violent impacts of waves that are either breaking or very close to breaking. Design formulae for estimating the magnitude of the impulsive pressures generated by breaking waves are presented, for example, in Ref. [8]. These relationships are largely derived from the results of laboratory tests rather than from an in-depth analysis of the fundamental mechanics. A review of the more theoretical aspects of wave impacts on walls is provided in Ref. [9]. Experimental scale model testing can assist with some of these issues, but the results are much more uncertain under extreme wave conditions due to scale effects. One of the most common difficulties of conducting experiments with WECs is the presence of scale effects: the hydrodynamics requires different model scales and the influence of the various effects is difficult to infer from small-scale experiments. This makes numerical modelling a particularly valuable tool in the development of WECs. For OWSCs, inspiration was found in a field apparently disconnected from the field of wave energy: the transport of liquefied natural gas (LNG) in LNG carriers, where extreme loading (slamming) can occur and damage the LNG tanks in extreme sea states. For nearly 10 years now, the LNG community has been developing a mathematical and computational modelling framework as well as an experimental framework for the analysis of wave impacts arising in LNG carriers [10, 11]. At least six physical phenomena were shown to be of importance during impact, ranging from hydroelasticity and liquid/gas compressibility to interfacial instabilities and phase transition, with change in fluid momentum being the most important one. Both in the ocean and in laboratory experiments, it has been observed that OWSCs can pitch seaward violently before the next wave crest arrives (see Fig. 3). We will see in Sect. 4, on the modelling of the wave climate, that highly energetic sites off the west coast of Ireland are prone to slamming of OWSCs. For OWSCs, wave impacts on the flap during extreme sea states could have serious structural implications, which need to be quantified. In particular, the following questions need to be addressed: What are the local forces and pressure distributions across the flap during wave impact from a large wave? What are the local forces and pressures induced by the incident waves at key geometric points on the flap structure? What is the effect of entrained air pockets between the flap structure and the colliding wave?

**Fig. 4:**
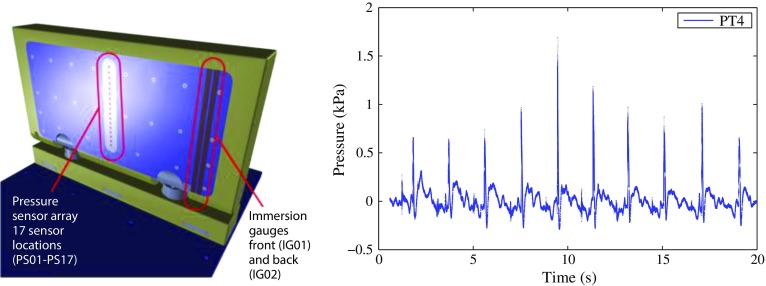
Experiments in Ecole Centrale Marseille: small-scale model (1/40) of the Oyster WEC, location of pressure sensors and immersion gauges. Pressure signal at one pressure transducer (PT4) showing the impulsive load every period

**Fig. 5:**
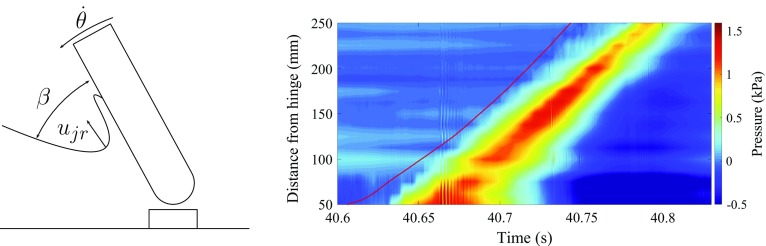
Sketch of the free surface and the evolution of a jet during slamming. Pressure footprint on the front face of the flap as a function of time, following, with a slight delay, the immersion gauge measurement of the tip of the jet (*red line*). The oscillations, which are visible between time $t=40.65$ s and $t=40.7$ s and between 5 cm and 10 cm from the hinge, are typical of compressible air pocket oscillations

We addressed the impact question through computational and experimental modelling at the local level of a single WEC.

### Experiments

The slamming of OWSCs was first observed during a set of 3-D experiments [13]. However, it was very difficult to understand the characteristics of the violent flow near the flap. Therefore it was decided to switch to 2-D experiments. As opposed to 3-D experiments which are difficult to visualize, 2-D experiments provide a better view to capture images of the slamming process. Moreover, 2-D experiments are easier for mapping the pressure on the flap. This pressure map is useful to gain insight into the slamming phenomenon.

The first 3-D experiments, conducted in Queen’s University Belfast in collaboration with APL using a small-scale model of Oyster (1 / 25), revealed high impulsive loads, associated with slamming events [13]. This study allowed identification of the different steps in a wave cycle leading to such slamming events, namely: (1) the flap is pushed towards the beach as the wave crest approaches; (2) the flap oscillates back seawards in the trough of the wave with the free surface lowering on the front face of the flap until it reaches a maximum dry-out when the flap is almost vertical; (3) the flap re-enters the water with a high angular velocity and the water level starts moving up the flap; until (4) the water is ejected at the top of the front face and the cycle starts again.

These observations showed that the impact is dominated by the re-entry of the flap in the wave trough and that it is the flap that impacts the wave rather than a classical wave impact. The slamming of OWSCs has, therefore, been related to water entry problems. The subsequent 2-D experiments, where the free surface is more easily tracked, showed similar trends thereby confirming the earliest observations (see Fig. 3). In the first set of 2-D experiments, conducted in Ecole Centrale Marseille [12, 14], only one pressure sensor was available, so a new set of experiments was conducted with a new small-scale Oyster model (1 / 40) [15] (Fig. 4).

The 2-D experiments emphasized the importance of the development of a jet in the pressure distribution along the flap, similar to a Wagner-type impact [16–18]. The pressure at a given transducer reaches a maximum when the jet root (defined in Fig. 5) passes in front of the sensor and the maximum pressure at that instant can be estimated from the jet-root dynamic pressure to a high degree of accuracy [15]:

1 $$\begin{aligned} p_\mathrm{max} = \frac{1}{2} \rho u_{jr}^2, \, \end{aligned}$$

where $\rho$ is the density of water and $u_{{jr}}$ is the jet-root velocity calculated from post-processed images. During a slamming event, the impulse pressure propagates towards the top of the flap along the jet root (see Fig. 5).

Even though the propagation of the jet is very similar to what happens in water entry problems, the initial stage of the impact is not always well represented by the dynamic pressure (1) and similarities with flip-through impacts [19] have been observed. In addition, although only bubble clouds and no big air pockets are observed (see Fig. 3), oscillations at the frequency of an air pocket can be seen in the pressure signals (see Fig. 5). These oscillations raise certain questions regarding the effects of compressibility that cannot yet be answered for this type of impact.

### Numerical simulations

In the process of developing the numerical tools, several difficulties were encountered; a major one was the handling of the mesh during large motions of the flap. One of the goals of the numerical simulations was to provide a full description of all forces acting on the device in extreme waves: inertial forces, drag forces, hydrodynamic radiation and diffraction effects. Scaling issues become more uncertain in extreme wave conditions.

CFD methods can take into account nonlinear effects naturally, e.g., flow separation, turbulence and wave impact, which may be important for predicting the hydrodynamic forces. CFD also can provide comprehensive flow details, and allow simulations at various scales, for various device shapes and wave conditions. These features make CFD an attractive method for studying wave-OWSC interaction problems. To handle the large motions of the flap, there are three computational techniques commonly employed: moving mesh methods, fixed mesh methods, and meshless methods. The guideline for selecting a particular approach is that its algorithm should be accurate, robust, and computationally inexpensive. In Refs. [12, 20], dynamic mesh methods were used to describe the flap motion and investigate the viscous effects and slamming on an OWSC. In Ref. [21], a model based on the immersed boundary method was developed to investigate wave interactions between waves and a modular OWSC. In Ref. [13], the smoothed particle hydrodynamics (SPH) method was used to investigate 2-D and 3-D slamming on an OWSC (see below). Another difficulty is the high computational cost of slamming simulations in 3-D. In order to reduce the computational cost while avoiding the re-reflection at the outer boundary, a “wavemaker-less” model with “relaxation zones” was developed to investigate the 3-D effects of wave slamming on an OWSC [22]. In addition, a hybrid model combining a Boussinesq model (FUNWAVE) and a finite-volume model was proposed to simulate, at affordable computational cost, some of the conditions experienced by the full-scale Oyster 800 device, incorporating real bathymetry at the deployment site [23].

**Fig. 6:**
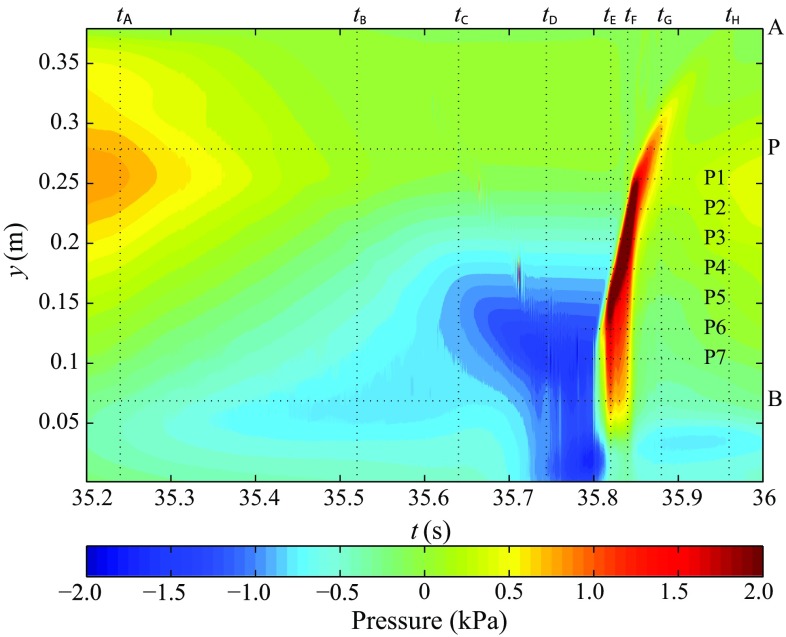
Time histories of pressure on the seaward face of the flap during an impact event, obtained by numerical simulations

With the numerical models, we first checked whether viscous effects played a role or not in the flow around the device (turbulence, vortex shedding) [20]. Intensive simulations demonstrated that vortex shedding from the flap is a short-lived, periodic phenomenon, and that viscous scaling effects are not an important issue for OWSCs. The continuous time–space distribution of the pressure on the flap surface [12] demonstrated the “slosh-type” character of the impacts and indicated the location of the strongest impact pressure on the flap (Fig. 6). In addition, the CFD results helped us to understand the re-reflection effects in the 2-D slamming experiments. The main discovery was that the slamming intensity could be enhanced or suppressed due to the re-reflection, depending on the wavelength and the distance between the wavemaker and the flap. Simulations of 3-D slamming events [22] showed the difference between 2-D and 3-D slamming. In 3-D slamming, water re-entry begins at the sides and focuses into the centre, thus enhancing the impact pressure there.

It was assumed that the flow was incompressible, noting that the experiments did not show much evidence of compressible effects. The elasticity of the WECs was not considered either. Wave impact is such a complex problem that it is not possible to consider all phenomena together.

We also tested meshless methods. In contrast to classical methods such as finite-volume methods, meshless methods do not need any grid or connectivity constraint between the computational nodes to simulate the domain; hence, they can model problems with large deformations such as wave interactions with OWSCs. Our group used the SPH method, which is a meshless, purely Lagrangian technique originally developed in 1977 [24–26]. It has subsequently been successfully employed in a wide range of problems [27].

**Fig. 7:**
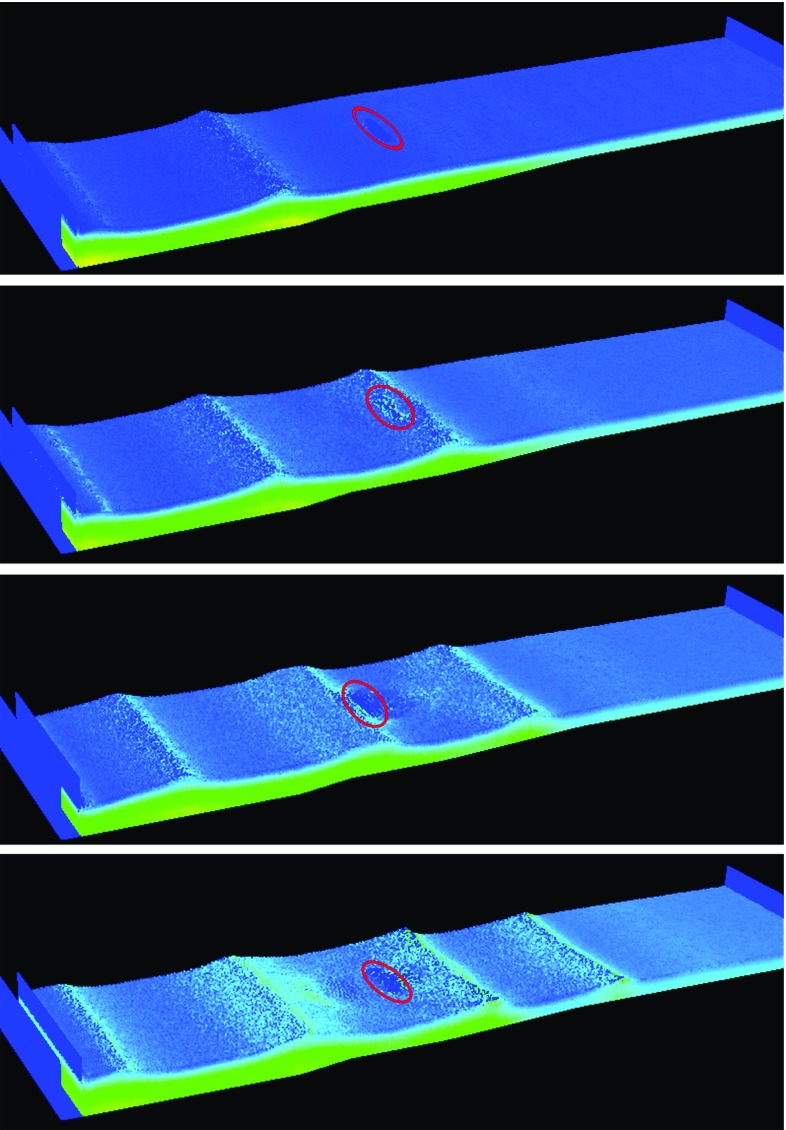
SPH simulation of wave interaction with an OWSC. Particles are coloured by their pressure, *blue* being low and *yellow* being high pressure. For clarity the OWSC position is highlighted by a *red circle*

In SPH, moving nodes (carrying field variables such as pressure and density) are defined as the “particles”and advected with the local velocity. Since the fields are defined only at a set of discrete points, to ensure differentiability, a continuous field is defined by interpolation kernels. In the weakly compressible SPH formulation (WCSPH), the fluid is assumed compressible with a large sound speed (such that the Mach number $M \approx 0.1$ and the density of the fluid typically varies by less than $1 \%$). The SPH method uses smoothing kernels to express a function in terms of its values at a set of disordered points. The smoothing kernel function (or weighting function) specifies the contribution of a typical field variable, *A*(*r*), at position *r* in space.

In order to model wave interactions with an OWSC, the SPH particles were initially placed on a grid of squares with initial spacing of $l_0 = 0.033$ m resulting in a total number of 3, 264, 668 particles [13, 28]. The SPH smoothing length was set to $h = 1.5 l_0$ and the boundary particles were placed with a spacing of  $l_0/3$. Like the finite-volume simulations described above, the SPH simulations were performed on ICHEC’s (Irish Centre for High-End Computing) Stokes supercomputer, which is an SGI Altix ICE 8200EX cluster with 320 compute nodes. The SPH simulations presented here used 72 processors and took ${\sim }70$ h for 13 s of physical simulation time.

**Fig. 8:**
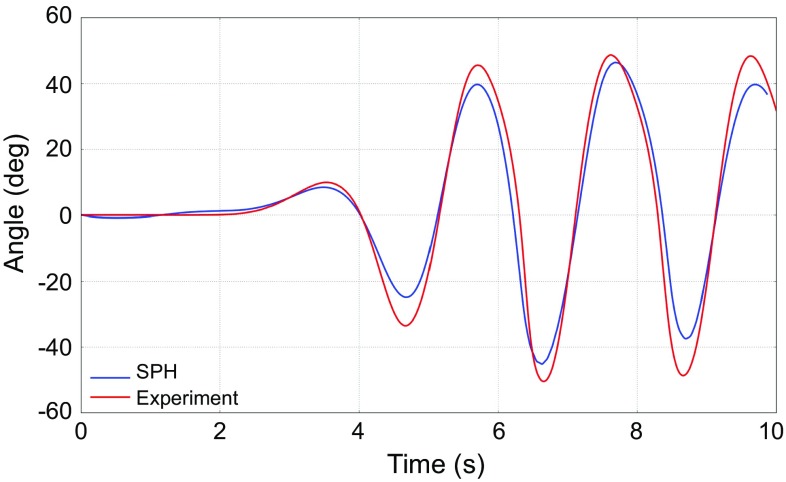
Comparison of numerical and experimental time histories of rotation angle of the OWSC

Figure 7 illustrates the simulation output of the entire wave tank. Waves progress from left to right past the OWSC at the centre of the images. It is crucial that simulations accurately predict the motion of the flap before any comparison of pressures is made. The simulated time variation of the flap angle shows good agreement with experimental data (Fig. 8).

**Fig. 9:**
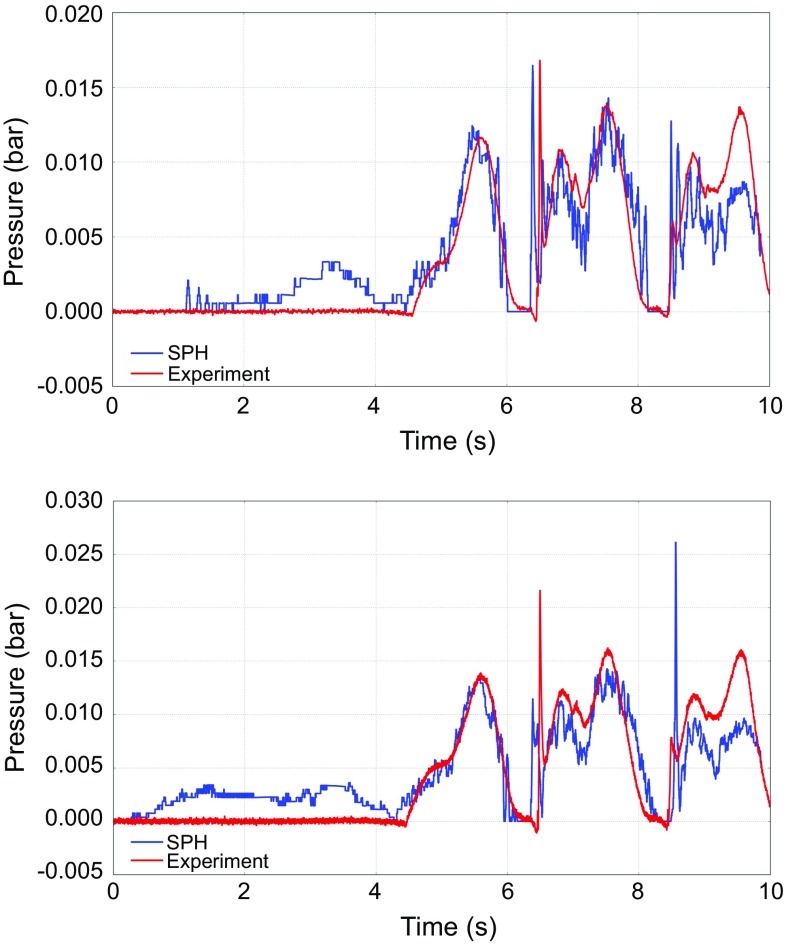
Comparison of the time history of pressure variations between numerical and measured values at two sensors on the flap (1 bar = 100 kPa)

Figure 9 presents the time history of the pressure exerted on two pressure transducers located on the OWSC. The model predictions are in good agreement with the experimental data. Slight discrepancies between numerical estimations of the maximum pressure peaks and the experimental data are due to the well-known stochastic nature of wave impacts which leads to scatter in the experimental results. In both cases, SPH simulations were capable of predicting the sharp pressure peaks. However, these peaks are stochastic, and therefore, their exact location in time and their peak value are not repeatable in the experiments. Therefore, slight discrepancies between the numerical values and experimental data are to be expected.

The 2-D and 3-D CFD simulations and experiments have greatly improved the understanding of slamming and viscous effects in a situation where only one OWSC is present. Next, we must consider arrays of OWSCs. However, it is clear that the cost of CFD would be prohibitive for arrays. In the next Section, we review alternative methods to model arrays.

## Arrays of wave energy converters

The commercial feasibility of wave energy demands a modelling environment that extends to multiple WECs. A single WEC, with a capacity comparable to a classic power plant (400 MW, say) is technologically impossible. Therefore, arrays of WECs, placed in a geometric configuration or farm, are needed. In a farm, WECs interact and the overall power absorption is affected. Determination of the optimal pattern of WECs in order to maximise power absorption is of major importance in the design of a wave farm, and this pattern would be expected to depend on the specific wave climate experienced at the site of interest (see Sect. 4). The fundamental modelling of arrays of WECs, which is based on linear wave theory, was presented almost 40 years ago [29, 30]. A comparison of the multi-scattering method, the plane wave method and the point-absorber approximation was presented in Ref. [31]. Analytic expressions for wave absorption by a periodic linear array were derived in Ref. [32]. A numerical code based on the boundary element method (BEM) was used in Ref. [33] to study the impact on the absorbed wave power of the separation distance between two WECs and the wave direction. BEMs are powerful and used extensively in the study of floating bodies. However, they are computationally expensive. An acceleration of the BEM code by a fast multipole algorithm was presented in Ref. [34]. However, the results were mixed because of the slow convergence of the expansions for large wavenumbers.

We could also have used a BEM code given that our group has developed an efficient 3-D BEM code over the years [35, 36]. However, we felt that the mixed results obtained in Refs. [34, 37] were not the best route to follow for the time being. We also thought of Boussinesq modelling. For example, a Boussinesq code with rectangular bottom-mounted (surface-piercing) structures has been used successfully in Ref. [38]. But the inclusion of structures in Boussinesq codes remains challenging. Instead, we decided to rely on analytical methods. Since no linear model existed for OWSCs, we had to derive such a model from first principles.

### Mathematical model of a single wave energy converter

**Fig. 10:**
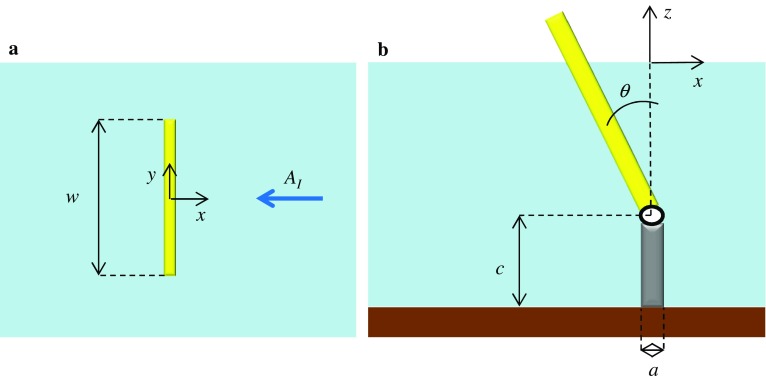
Geometry of the 3-D analytical model of an OWSC. The incident waves are travelling from *right to left*. From Ref. [1]

A mathematical model has been developed to study the behaviour of an OWSC in a channel, noting that, during laboratory tests in a wave tank, peaks in the hydrodynamic actions on the converter occurred at certain frequencies of the incident waves. This resonant mechanism is known to be generated by the transverse sloshing modes of the channel. However, the extent to which resonance would affect the behaviour of the device was not known. We developed a semi-analytical model to better understand the effect of such resonant peaks on the power production of the device. The geometry is shown in Fig. 10. Within the framework of a linear inviscid potential-flow theory, application of Green’s theorem yields a hypersingular integral equation for the velocity potential in the fluid domain. The solution is found in terms of a fast-converging series of Chebyshev polynomials of the second kind. The physical behaviour of the system was then analysed, showing sensitivity of the resonant sloshing modes to the geometry of the device. Our analytical results agree very well with available experimental observations, see Refs. [39, 40]. Our model shows that the initial two-dimensional motion of the incoming waves in the channel shatters into a series of three-dimensional sloshing waves. Each sloshing mode resonates at a specific wavelength $\lambda _n$, namely

$$\begin{aligned} \lambda _n = b/n,\quad n=1,2,\dots , \end{aligned}$$

where *b* is the channel width. We showed that when a sloshing mode resonates, its energy becomes trapped near the flap and the system efficiency increases, up to 80% for a simple device similar to the Oyster WEC. The model was then modified to deal with an OWSC in the ocean (no channel) [41]. We showed that the behaviour in the ocean is substantially different from the one in the channel, because of the different patterns of the radiated waves in the open ocean configuration. The results showed that the influence of the lateral walls in the channel resulted in a 10% increase of the wave torque acting on a device resembling Oyster 1, with respect to the open ocean scenario. This shows that extra care should be taken when one uses the results obtained in a wave tank to predict the behaviour of the OWSC in the open ocean. A detailed analysis of the channel effect revealed that a blockage ratio greater than 20% could significantly affect the performance of the device in the channel with respect to its behaviour in the ocean [41]. The capture factor of a single Oyster WEC has been given for six different sea states at the European Marine Energy Centre (EMEC) test site [42].

### Model for arrays of wave energy converters

**Fig. 11:**
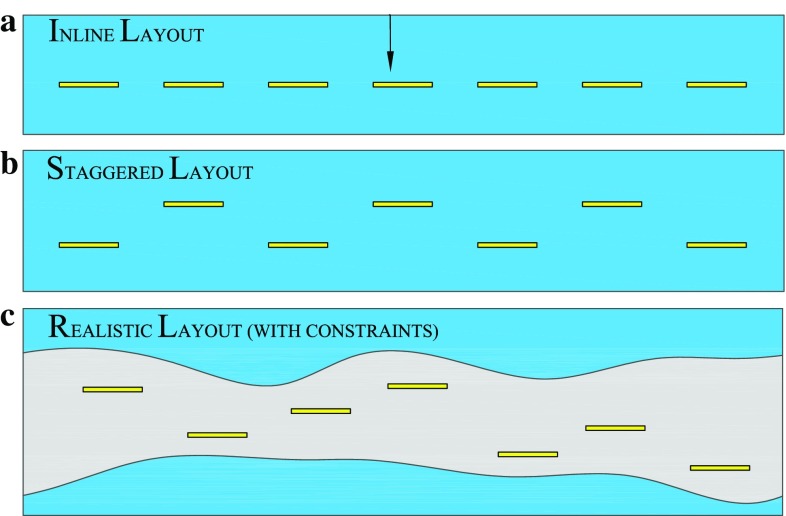
Finite array of OWSCs. **a** Inline layout (2-D symmetry and uniform spacing); **b** staggered layout (1-D symmetry and uniform spacing); **c** a realistic wave energy farm layout

The modelling of wave farms falls within a framework that must include wave interactions, wave reflection and diffraction phenomena. We analysed the interaction of waves with an array of WECs and determined the performance of the array with respect to identifiable design parameters. In the simple case (analytically convenient) of an inline periodic array, comprising an infinite number of OWSCs, the relevant parameter of interest is the single spacing parameter [39, 40]. The first mathematical model for a finite number of OWSCs was restricted to an inline layout of up to three OWSCs [43], and it was shown that the inline configuration (see Fig. 11a) exhibited near-resonant behaviour, similar to the resonant characteristics of an OWSC in a channel which can enhance the performance at specific frequencies. Later, a semi-analytical approach was developed for an array with an arbitrary number of OWSCs and arbitrary layouts. This model facilitated the analysis of practical layouts of WECs, and it was shown that a staggered configuration can lead to a better performance than an inline one in random seas.

Arrays of flap-type systems have also been investigated in other contexts, e.g., Venice gates (see Refs. [44, 45]), and the possibility of using the latter for harnessing energy has been recently explored [46]. In some special designs of WECs, an individual device can itself comprise an array of smaller oscillating components. For example, the novel modular OWSC concept [47] consists of multiple flap-type components. The motivation for such a design was to address a shortcoming in the original OWSC configuration in the form of large wave loads acting on its bottom foundation. The idea is to distribute the wave loading on several flaps to mitigate the detrimental effects. However, the new design leads to more complex hydrodynamic interactions amongst the individual flaps of the device. We identified multiple resonating frequencies of the system which could lead to large oscillations of the flaps. We observed that the performance of the device is strongly dependent on the PTO characteristics of the individual flaps due to the nature of the hydrodynamic interactions. The new concept provides flexibility in terms of tuning the individual components of the system. However, they need to be optimized together in order to maximize the power captured by the device as a whole. For PTO characteristics (per unit width) similar to that of the original design, the total powers captured by the two systems are comparable.

A key challenge in the planning of wave energy farms is the identification of optimized array layouts. Although several mathematical models are available for the evaluation of hydrodynamic interactions, their computational costs are prohibitive for optimization purposes. The existing work on array optimization was limited to small arrays [48, 49], and in most cases constrained by symmetrical layouts and/or uniform spacings, which reduced the dimension of the optimization problem. For example, the inline layout (see Fig. 11a) is 1-D with uniform spacing and, therefore, has one design parameter, while a staggered array (see Fig. 11b) is symmetric and uniformly spaced with two design parameters. A realistic array (see Fig. 11c) could have an arbitrary arrangement with constraints imposed by operational, economical and natural (bathymetric) limitations. We introduced a new approach for array optimization based on machine learning techniques [50], which, to our knowledge, enabled optimization of large arrays for the first time. The main idea is to develop a cheap surrogate model for the performance function of the array, which then is used for optimization. The method first uses a statistical emulator [51], based on Gaussian processes (see Ref. [52]), to predict the performance in small clusters. The original array is then formulated in terms of small clusters and a meta-model is derived for the whole array. The high dimensional optimization is then performed using a custom genetic algorithm. The simplification of interactions is facilitated by an important, yet practical, assumption that any particular WEC is largely influenced by only its nearest WECs. We optimized layouts for 40 WECs under different constraints, but the approach would work equally well for even larger arrays. The performance of arrays of Oyster WECs has been evaluated for the most probable sea-state at the Isle of Lewis in Scotland [43, 50] —see Sect. 4 on wave climate.

### Semi-analytical model for a single wave energy converter including viscous effects

Semi-analytical models [39, 41, 43, 50, 53] neglect the effects of viscous dissipation. However, experimental wave tank tests and CFD simulations have shown that flow separation occurs at the edges of OWSC flaps [20]. Due to the time-consuming nature of wave tank testing and CFD simulation, it is inefficient to use these tools to quantify the effect of this viscous dissipation on the hydrodynamic performance of OWSCs in varieties of sea conditions and device orientations.

An alternative to CFD is to use a BEM approach combined with a Morison-type drag law [54]. Such an alternative is suited to bodies whose characteristic dimension $w^\prime$ is small compared to the incident wavelength $\lambda ^\prime$, i.e., for small diffraction parameters: ${ Kl}=2\uppi w^\prime /\lambda ^\prime \ll 1$; in addition, the Keulegan–Carpenter number ${ KC}=2\uppi A^\prime _I/w^\prime \gg 1$, where $A^\prime _I$ is the amplitude of the incident wave, should be large. Generally speaking, the use of Morison’s equation is permitted when ${ KC}>6$, and ${ Kl}<1$ [55]. Typically, neither of these hold in the current mathematical models of flap-type WECs, with ${ Kl}=O(1)$ and ${ KC}\ll 1$ due to the assumption of linearity.

Another alternative is to modify the inviscid theory in regions of the fluid domain where the effects of viscous dissipation are non-negligible [56]. In the case of an OWSC, this is near the edges of the flap [20]. Such an approach was adopted in Ref. [56] in a study of the free surface in a moonpool. In Ref. [56], a control surface was defined from the moonpool’s sharp edge down to the seabed, across which a pressure discharge is imposed. The pressure discharge law assumes a functional relationship between the pressure drop and the local flow velocity, which characterises the effects of dissipation. It is shown that such an approach eliminates unphysical spikes in the resonant free-surface of the moonpool (predicted by inviscid theory).

**Fig. 12:**
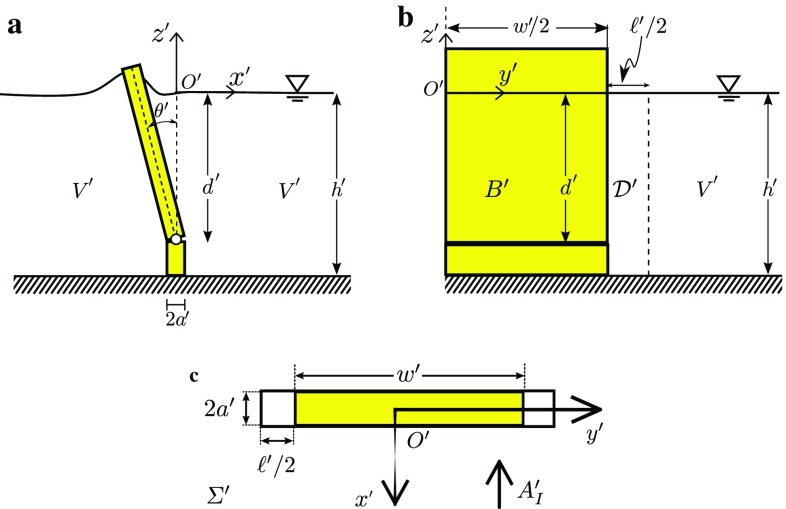
Dissipative surfaces: A bottom-hinged OWSC in a water depth $h^{\prime }$, hinged at a depth $d^{\prime }$ beneath the water’s free surface, with dissipative surface $\mathcal {D}^{\prime }$ extending from the OWSC’s edges. **a** Side, **b** front, and **c** plan view. Waves are incoming in the negative $x^{\prime }$ direction

The pressure discharge law typically takes the form of a linear [56–58] or quadratic [59] function of the local flow velocity. In addition, an effective linear law may be used in place of the nonlinear one using the Lorentz principle of equivalent work [60]. In the past, numerical models of OWSCs have used effective linear [54, 61] and quasi-linear [62] drag laws in place of the standard Morison (quadratic) drag law [54]. In each of Refs. [56–58, 63], it is assumed that the pressure drop across the screen/breakwater due to viscous effects is a linear function of the local flow velocity.

More recently, in Ref. [64], the effect that viscous dissipation has on an OWSC is examined by modifying the semi-analytical theory of Renzi and Dias [39] to include the effects of viscous dissipation near the edge of the flap. This is achieved by applying an effective pressure discharge

$$\begin{aligned} \varDelta P^{\prime }=f({v}^{\prime }_{n}), \end{aligned}$$

in the vicinity $\mathcal {D}^{\prime }$ of the edges of the flap, where $\varDelta P^{\prime }$ denotes the difference in the pressure $P^{\prime }$ from the left to the right side of the flap/dissipative surface in the wave direction (see Fig. 12). The equation of motion of the flap is then solved in the frequency domain, and the solution is used to conduct a parametric analysis of an OWSC for a variety of environmental conditions and device dimensions. We conclude that the effects of dissipation are to reduce the peak values of the hydrodynamic quantities, and that the dependence of the hydrodynamic quantities on the dissipation is generally weak when considering the environmental conditions typically experienced by existing OWSC designs. The effects of dissipation are strongest near peaks in the hydrodynamic quantities and for long-period waves. The effect of dissipation is negligible for short-period waves. The conclusion in Ref. [64] that viscous drag is more important for narrow flaps, and that the effects are amplified for long-period waves is in agreement with existing numerical and physical modelling data [20].

The 2-D and 3-D analytical and computational models have greatly improved our understanding of arrays of OWSCs. However, arrays must be placed in wave energetic areas and at the same time be accessible for maintenance. In the final section, we review wave climate assessment with the aim of addressing a range of critical dependent issues related to wave energy applications.

## Wave climate assessment for wave energy systems off the coast of Ireland

It is essential to understand the wave resource for at least four reasons: (1) one needs to know what the average wave power is in the area where one wants to deploy WECs; (2) one needs to know the waves in more detail if one wants to use control to optimize the efficiency of the WECs [65–70]; (3) one wants to know when access to the WECs will be possible in case maintenance is needed [71, 72]; and (4) one wants to ensure the WECs can survive the more extreme wave conditions expected in the area of deployment. Ocean waves are created by wind and then propagate freely in the ocean, forming swells. When they enter coastal waters, the limited water depth affects the amplitude and direction of ocean waves. Surprisingly, wave properties in shallow water are not yet fully understood. Better descriptions and measures of local fluid particle velocities in shallow water waves, as well as increased knowledge on waves close to breaking, are needed [61]. Over the past two decades, the development of increasingly accurate and efficient numerical models of non-linear surface waves has been a continuous challenge to the ocean and coastal engineering communities. Many types of wave and storm surge models have been developed to represent conditions under major storms, either for real-time forecasting or later hindcasting. These models are theoretically applicable—given suitable forecast wind fields—for spatial scales spanning at least four orders of magnitude: from ocean basin scales (thousands of kilometres) down to coastal scales (hundreds of metres). In practice, however, the computational efficiency of existing models severely limits the range of spatial scales accessible [73]. Moreover, given models with fixed parameters may perform well on a given storm and not so well in other cases. There are a variety of classical spectral wave models available to scientists and engineers, including SWAN (Simulating WAves Near-shore) [74] and WAVEWATCH III [75] in the spirit of the WAM model [76]. In these models, one solves the random phase spectral action density balance equation for wavenumber-direction spectra. The implicit assumption of this equation is that properties of the medium (water depth and current) as well as the wave field itself vary on time and space scales that are much larger than the variation scales of a single wave. The models are being constantly improved, and so we used the state-of-the-art versions to assess the nearshore wave resource of Ireland [71, 72, 77, 78].

We validated recent versions of WAVEWATCH III [4.18] along the west coast of Ireland where wave data are available from several buoys. Of interest also is the question of whether the use of full-spectral third-generation wind-wave models such as WAVEWATCH III is sufficient for nearshore wave prediction, or if it is necessary to couple such spectral wave models with shallow-water type models in very shallow water where wave transformations and wave breaking are important. There is no consensus at the present time (see for example Ref. [79]). However, recent improvements in numerical wave models such as the development of better numerical methods, the inclusion of currents, and water levels, and the better parameterization of nearshore wave processes have enabled the increasingly accurate modelling of coastal regions (see for example Ref. [80], or the WAVEWATCH III Development Group [75]). We developed fruitful collaborations between atmospheric modellers and wave modellers. Indeed, the choice of wind forcing, such as from the European Centre for Medium-Range Weather Forecasts (ECMWF), is as important as the choice of the wave model.

Met Éireann (the Irish Meteorological Service) developed techniques to produce optimal wind forecasts for driving a regional version of the WAVEWATCH III model, and to improve the prediction of wind and wave conditions in the nearshore. The combination of state-of-the-art atmospheric and wave models provided enhanced wave climate predictions, with an emphasis on preferred geographical locations for OWSCs in Ireland. In Ref. [72], a 14-year hindcast was carried out to create a wind and wave atlas for Ireland. The winds were dynamically downscaled from ERA-Interim reanalysis to a 2.5 km horizontal resolution and 65 vertical levels using the HARMONIE-AROME configuration of the shared ALADIN-HIRLAM system (HARMONIE-37h1.1) [81, 82]. For the wave hindcast, we used WAVEWATCH III on a triangular unstructured grid with resolution ranging between 10 km offshore and 225 m in the nearshore, forced by the downscaled HARMONIE 10 m winds and ERA-Interim wave spectra.

The wind and wave hindcasts were thoroughly validated against available buoy data, including wave buoys in nearshore locations and coastal synoptic stations. Significant wave heights ($H_s$) and winds from hindcasts were compared against altimeter data from the CERSAT database at Ifremer. An improvement in the wind and wave validation compared to an ERA-Interim driven hindcast in Ref. [77] was found, particularly in coastal regions where the orographic affects of bays, islands and coastline features were more accurately resolved.

**Fig. 13:**
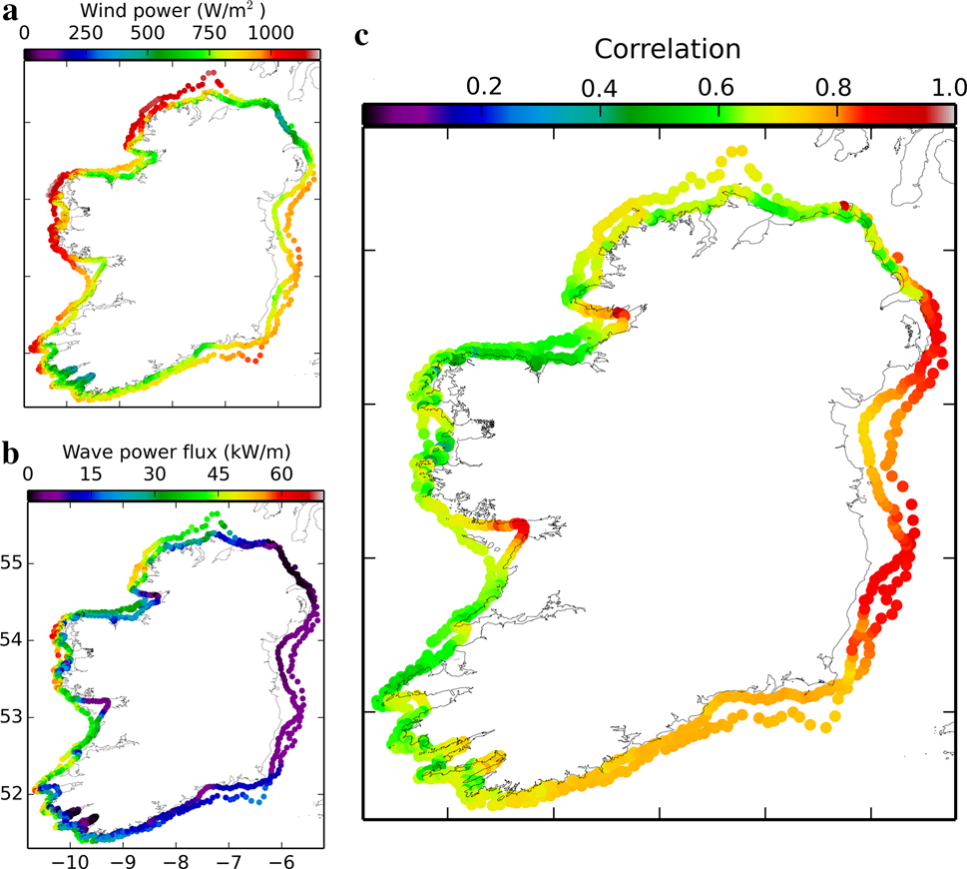
Complementarity of the wind and wave energy resource on the 30 m and 60 m bathymetric contours around Ireland, adapted from [72]. **a** Mean annual wind power at the 100 m height level (W/m$^2$); **b** mean annual wave power flux (kW/m); **c** correlation coefficient between the wind and wave energy

The study examined the complementarity between the wind and wave energy resource around the coast of Ireland. Joint wind and WEC farms could remove some of the high frequency variability of these renewable energy sources, which can create problems integrating these energy sources into the power grid. This is the case, for example, when there are energetic waves but little wind or vice versa. The focus was on the complementarity without focusing on any particular technology, to find suitable locations for joint wind-wave farms. This could improve the viability of future WEC deployments in such nearshore regions. Wave and wind hindcasts were interpolated to points on the 30 m and 60 m bathymetric contours, as can be seen in Fig. 13. The lower the level of correlation, the greater the complementarity between the two resources. Along the western seaboard, the lower correlation values indicate a higher occurrence of swell waves not generated by the local wind conditions (i.e., higher complementarity for a joint wind-wave farm), than on the eastern seaboard, where wind-seas dominate.

**Fig. 14:**
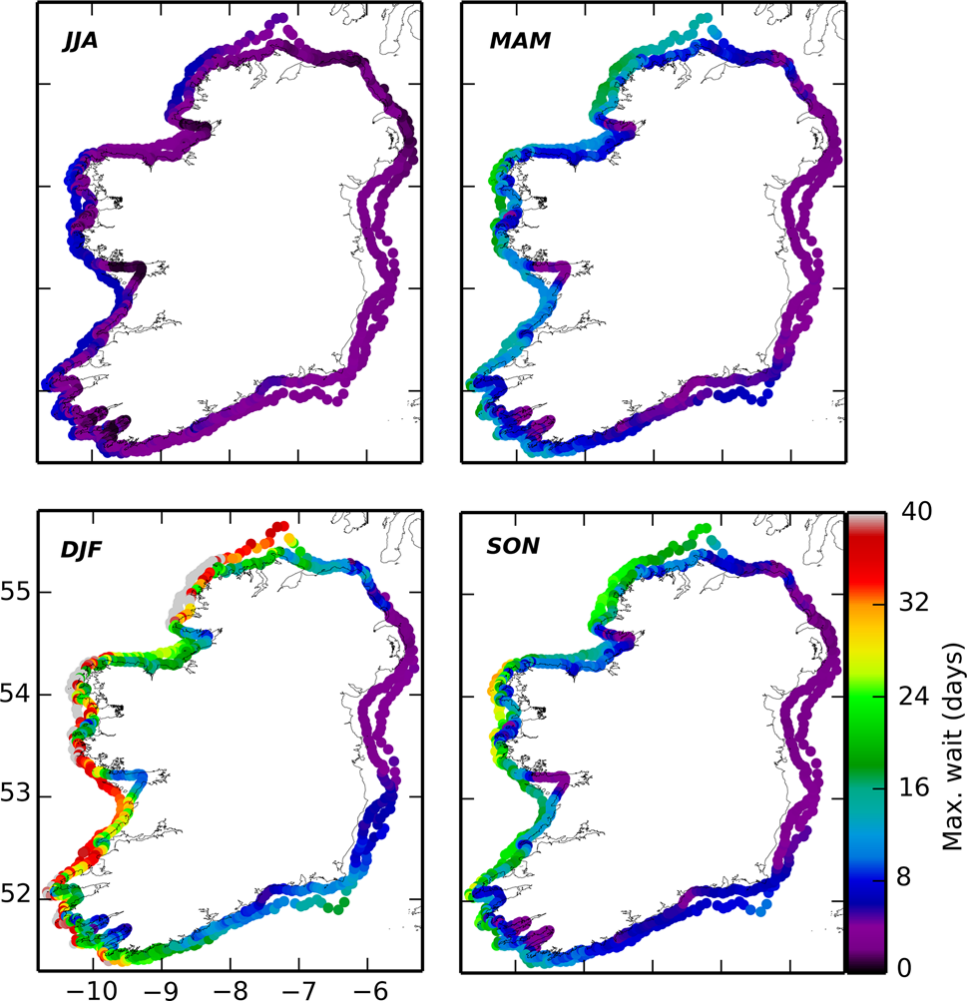
Seasonal weather window analysis for accessibility on the 30 m and 60 m bathymetric contours around Ireland. Average of the maximum waiting time for a weather window of at least 12 h duration satisfying the following criteria: (1) wind speed is less than 16 m/s; (2) $H_s$ is less than 2 m; and the peak wave period is less than 13 s. JJA—June, July, August; MAM—March, April, May; DJF—December, January, February; SON—September, October, November

Another important concern for marine operations is site accessibility for installation and maintenance. In order to assess weather windows around the coast of Ireland, and gain insight into the kind of operational planning required to maintain such WECs, we created sample criteria (for a generic vessel) to estimate accessibility on the 30 m and 60 m bathymetric contour [72]. As can be seen in Fig. 14, long waiting times were found for accessibility to sites in winter along the western seaboard (>40 days in some north–west regions)—unfortunately, a common experience for most marine operators.

A major limitation for the development of WEC farms is the issue of survivability. A combination of large swell and/or extreme locally-generated sea waves could lead to slamming (see Sect. 2), and, consequently, damage or destroy WECs. Ireland has a long history of extreme waves [83]. $H_s$ values of over 15 m are regularly recorded by the Irish Marine Buoy Network, by buoys located off the west coast of Ireland. Several maximum individual wave heights (trough to crest) greater than 20 m have also been measured by the buoy network. Recent studies have emphasized how extremes vary spatially off the west coast [84, 85]. Large scale atmospheric oscillations or teleconnections, such as the North Atlantic Oscillation, can also influence the likelihood of extreme wave events occurring, and more generally, the seasonal wave climatology and energy extraction potential of the North Atlantic for WECs [77, 86, 87].

**Fig. 15:**
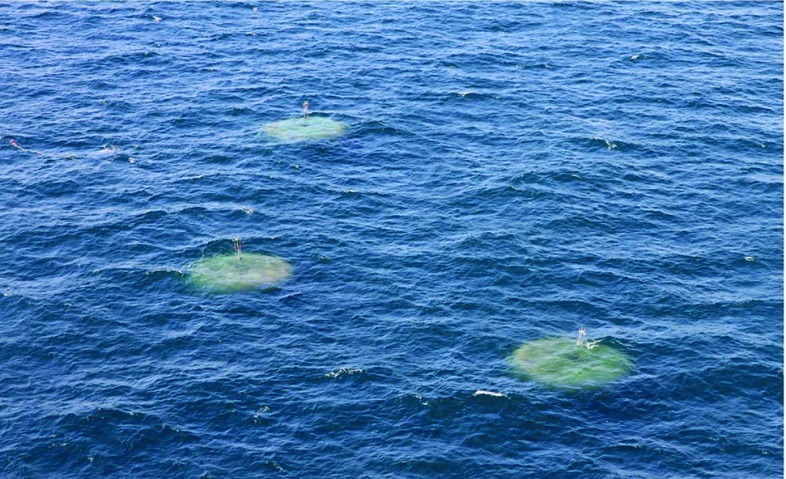
An array of three CETO-5 units in operation. The CETO WEC concept is developed by Carnegie Clean Energy

When planning long-term WEC installations, one must also consider the potential impact of global climate change on the marine resource. An ensemble of wave climate projections was carried out for Ireland to investigate how the waves, wind climate and storm tracks over Ireland and the North Atlantic might change towards the end of the century [78, 88], driven by EC-Earth wind and ice-fields [89]. Although a small overall decrease in the mean annual $H_s$ was found, evidence for changes in wave extremes were less robust—indicating that access for operational maintenance and survivability will continue to be an issue for WEC installations into the future.

## Concluding remarks

Wave energy is still at its infancy. However, remarkable progress has been made in the development of analytical and computational models for wave energy systems. Lessons must be learned from the recent failures of WECs and WEC companies. Fortunately, a few companies over the world are making progress towards making wave energy a reality. In the introduction, we mentioned AW Energy, which is developing the WaveRoller OWSC. Another company is Carnegie Wave Clean Energy Ltd, which is developing the CETO WEC. CETO is a fully submerged point absorber device that converts ocean swell into zero-emission renewable power and desalinated freshwater. Extensive numerical studies have been carried out on the CETO device. A prototype scale test of three of CETO units was installed recently and operated along the west coast of Australia as part of the Perth Wave Energy Project (PWEP) (see Fig. 15).

Future WEC deployments will be required to survive in harsh ocean environments. Recent developments in WAVEWATCH III concerning an improved modelling of extremes [90] will further enable an understanding of the most extreme operational wave loads.

Recent work has shown that WAVEWATCH III spectra can be successfully coupled to the full Navier–Stokes (or Euler) equations to generate extreme waves in intermediate depth water or even shallow water [91]. This type of coupling can only be performed in small areas of the ocean because of the high demand on CPU resources, but is perfectly suited to study a small area in the neighborhood of a WEC.

Although the number of studies taking into account a coupling between the atmosphere, the ocean and waves is still limited, such fully integrated Earth System models offer a promising means of providing a better understanding of the variability of the climate and of the wave climate.

Another issue of importance for future deployments of WECs is the inclusion of future wave information [66]. This is a key issue for the real-time control of WECs. Short-term wave forecasting is still a largely open question, even if progress has been made recently [67, 68]. One of the pressing questions is: How are free-surface evolutions best synthesized from wave spectra for power production assessment [69, 70]?
